# Supplementary material for: Intercellular adhesion boots collective cell migration through elevated membrane tension
Source: Nat Commun. 2025 Feb 12;16:1588. doi: 10.1038/s41467-025-56941-4 (PMC11822051; doi:10.1038/s41467-025-56941-4)
Supplement: Supplementary file 1 — Supplementary Information [file 41467_2025_56941_MOESM1_ESM.pdf]

Supplementary Information for:

Intercellular adhesion boots collective cell migration through elevated membrane tension

Description Of Additional Supplementary Files

- Supplementary Movie Legends 1-7
- Supplementary Figures 1-8
- Supplementary Table 1
- Supplementary Code 1-4

SUPPLEMENTARY MOVIE LEGENDS

Supplementary Movie 1: Cellular migration for wound-healing assay of MDA and MCF-7 cells. Frame rate (20 fps, with 20 frames per second). Scale-bar: 100 µm.

Supplementary Movie 2: Cellular migration for wound-healing assay of Cph1-PM-MDA cells under far-red and red light. Frame rate (20 fps, with 20 frames per second). Scale-bar: 100 µm.

Supplementary Movie 3: Single-cell tracking of individual cell nuclei of Cph1-PM-MDA cells under far-red or red light. Frame rate (20 fps, with 20 frames per second). Scale-bar: 100 µm.

Supplementary Movie 4: Cellular migration for wound-healing assay of Cph1-PM-MDA cells under red light for 6 hours and then under far-red light for 12 hours. Frame rate (20 fps, with 20 frames per second). Scale-bar: 100 µm.

Supplementary Movie 5: Cellular migration for wound-healing assay of Cph1-PM-MDA cells under far-red light for 6 hours and then under red light for 12 hours. Frame rate (20 fps, with 20 frames per second). Scale-bar: 100 µm.

Supplementary Movie 6: Cellular migration for wound-healing assay of Cph1-PM-MDA cells under far-red or red light in the presence of VU. Frame rate (20 fps, with 20 frames per second). Scale-bar: 100 µm.

Supplementary Movie 7: Cellular migration for wound-healing assay of Cph1-PM-MDA cells under far-red or red light in the presence of Oleic acid. Frame rate (20 fps, with 20 frames per second). Scale-bar: 100 µm.

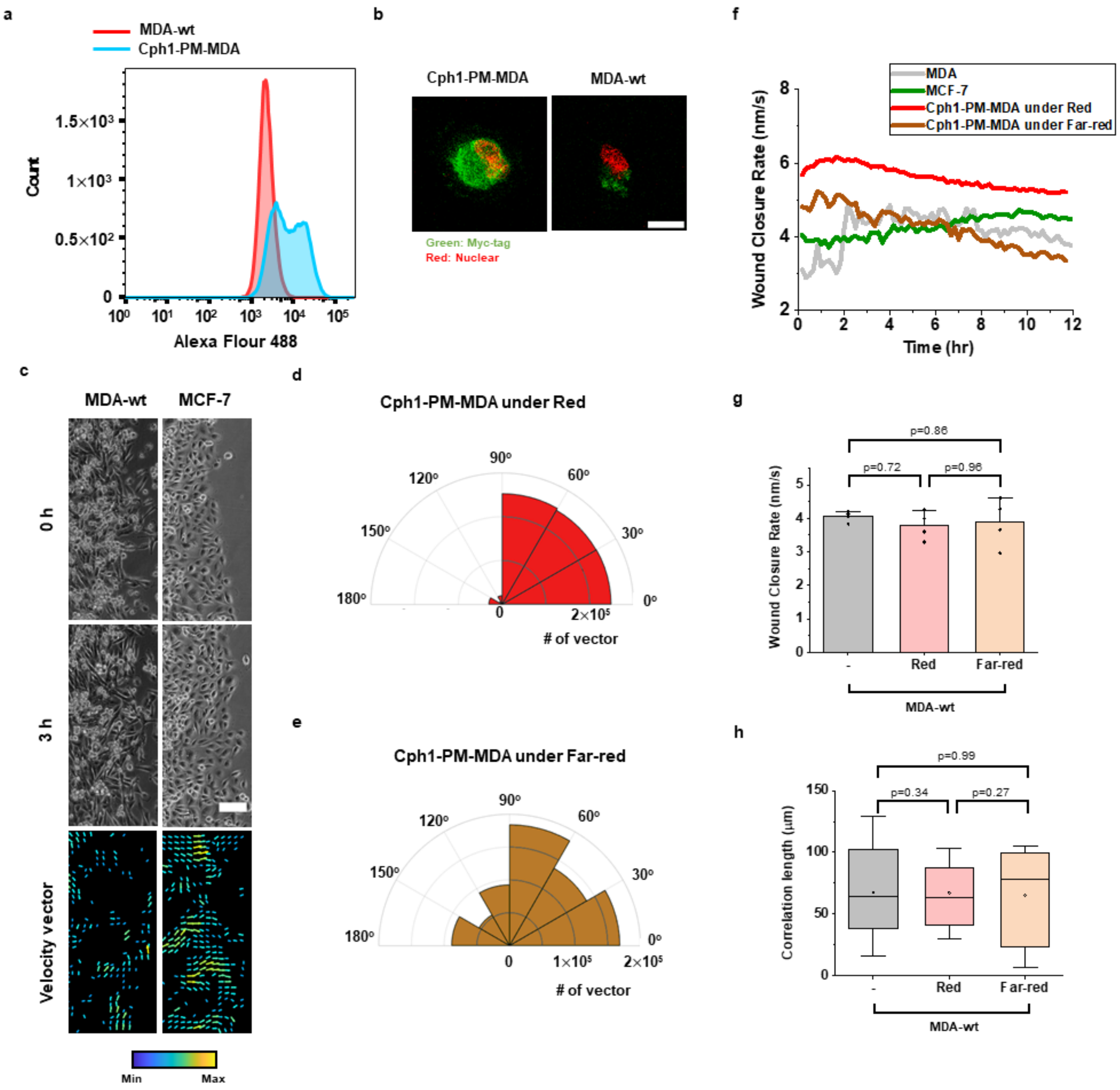

Supplementary Figure 1. Immunostaining of Cph1-PM-MDA and migration data for MDA-wt and MCF-7. (a) To probe for the presence of Cph1 at the cell surface, we immunostained live cells without permeabilization. Using flow cytometry, we compared the Cph1 signal of MDA-wt and Cph1-PM-MDA. **(b)** After cells attached, live cells were immunostained without permeabilization. Scale bars, 10 μm. **(c)** Wound closure rate of MDA-MB-231 (MDA) and MCF-7 cells. Scale bars, 100 μm. **(d,e)** Migration angle of velocity vectors of Cph1-PM-MDA under red (d) or far-red (e) light. **(f)** Velocity histograms depicting cellular migration patterns for MDA and MCF-7 cells. **(g)** Average wound closure rate of parental MDA without light (n=4, 4 biological replicates), under red light (n=4, 2 biological replicates), and under far-red light (n=4, 2 biological replicates). **(h)** Correlation length denotes the persistence length of the velocity vector components of MDA-wt without light (n=3443, 4 biological replicates), under red light (n=1769, 2 biological replicates), and under far-red light (n=1225, 4 biological replicates). Data in (g, h) were analyzed by one-way ANOVA with Bonferroni's multiple comparisons test. Bar plots (g) are denoted as the mean with standard deviation. Box plots (h) present the median and 25th and 75th percentiles, and the lower and upper boundaries of whiskers show the 5th and 95th percentiles. Data source and statistical details are provided as a source data file.

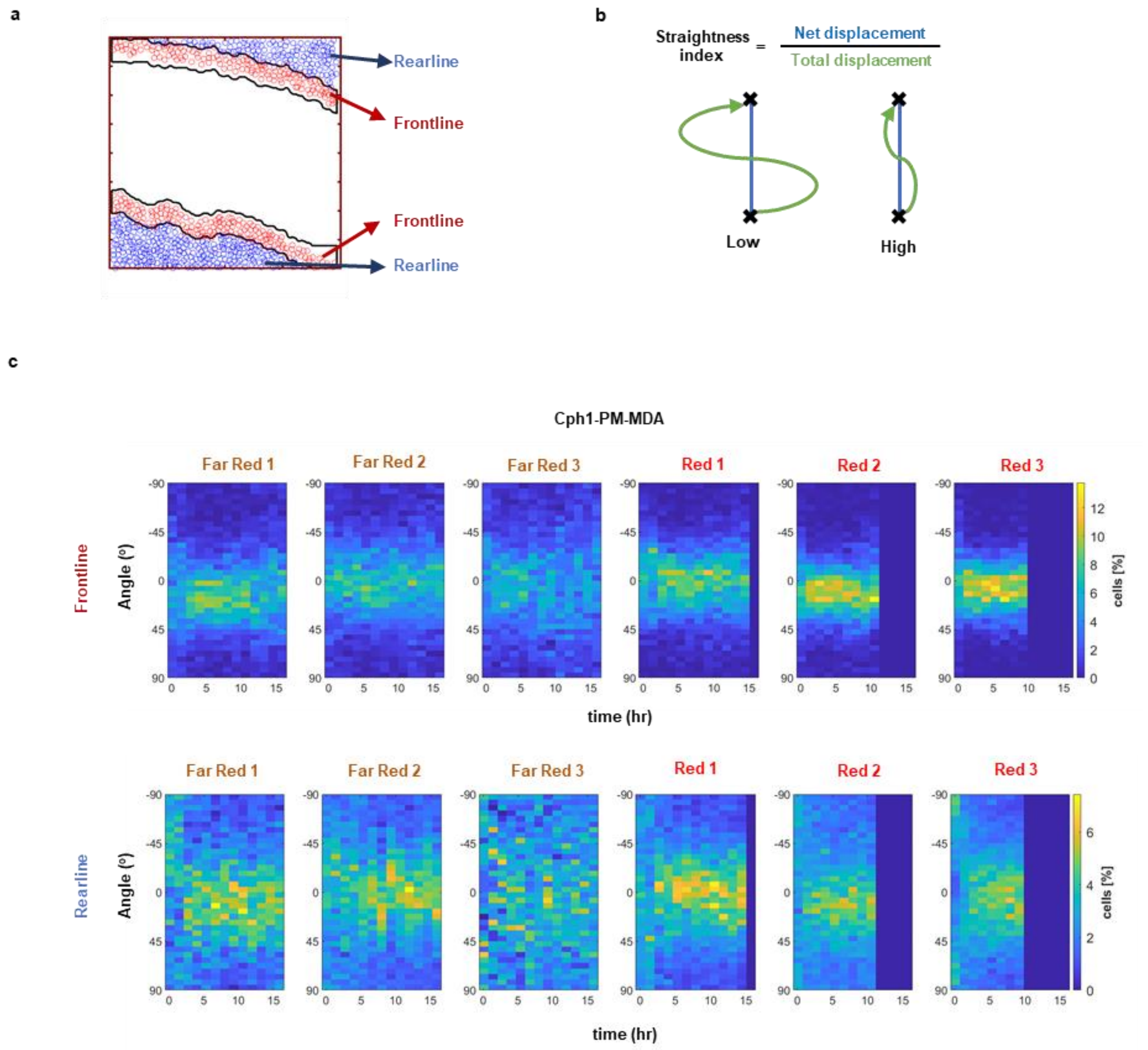

Supplementary Figure 2. Analysis of single-cell tracking data. (a) The regions were divided into frontline (red, consisting roughly of the first 3 cells lines at the edge) and rearline (blue, composed of all the remaining cells). **(b)** Schematic representation of straightness index. Straightness index is calculated by dividing the net displacement into total displacement. **(c)** The plots show the migration angle of individual Cph1-PM-MDA cells in the frontlines (top panels) and rear lines (bottom panels) under red (right panels) and far-red (left panels) light. Individual numbers indicate the technical replicates. Color code is uniformly scaled for all experiments at the frontline (top panels) and at the rearline (bottom panel), as indicated to the right.

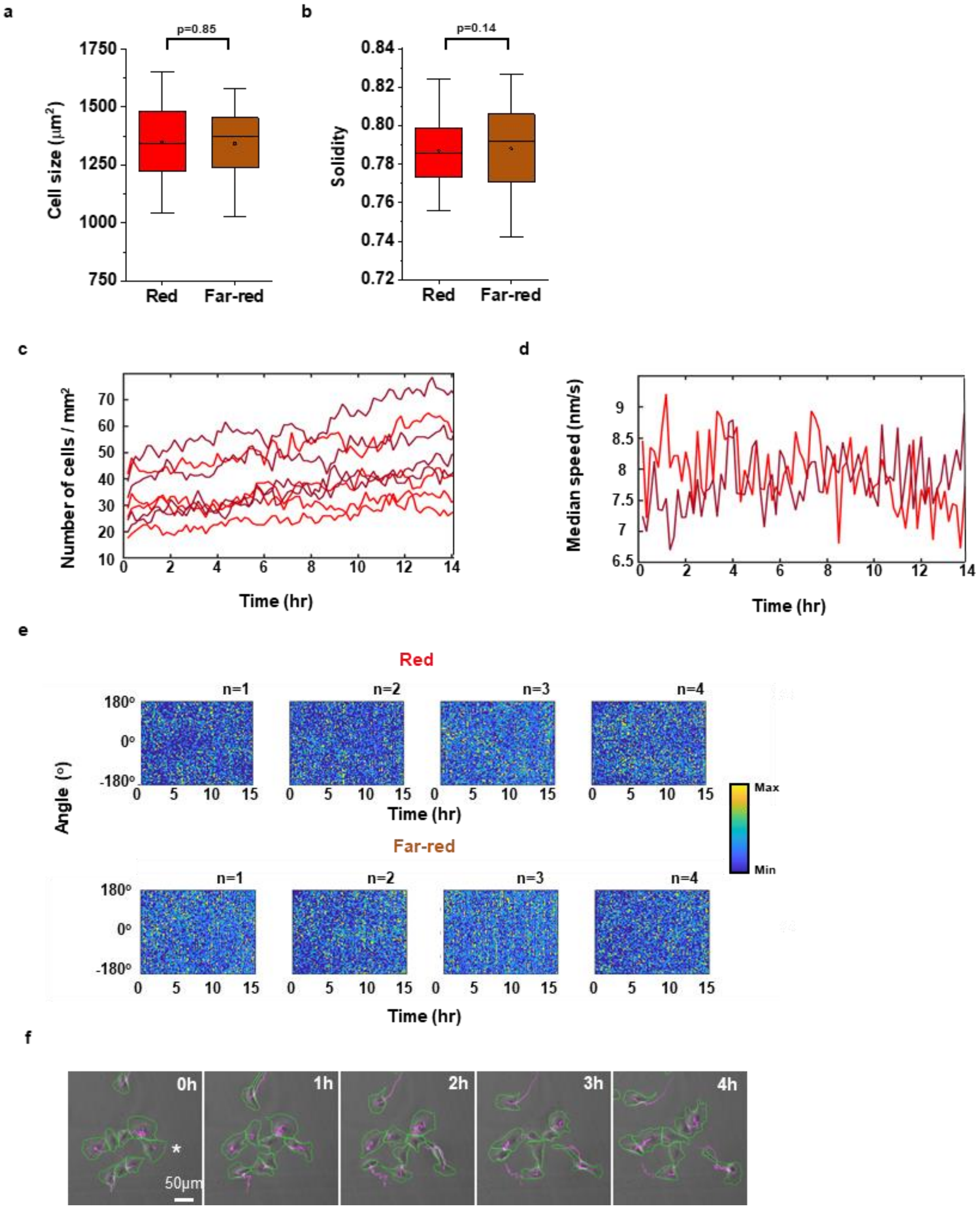

Supplementary Figure 3. Activation of Cph1-PM does not alter single-cell dynamics of Cph1-PM-expressing cells. (a, b) Shape analysis of sparsely cultured Cph1-PM-MDA cells exposed to red (red) and far-red (violet) light shows no significant differences in cell size (a) and solidity (b) between red and far-red light (n=384 from 2 biological replicates; two-tailed, two-sample Mann-Whitney test). **(c)** Growth rate of sparsely cultured Cph1-PM-MDA cells exposed to red (red) and far-red (violet) light. **(d)** Migration analysis of Cph1-PM-MDA cells exposed to red (red) and far-red (violet) light (n=4). **(e)** Migration angle of individual Cph1-PM-MDA cells in low cell concentration under red and far-red light. For better visualization, the color code in each experiment is individually set to cover the whole range (min-max). **(f)** Brightfield image of Cph1-PM-MDA cells overlaid with cell shape mask (green) and motion tracks over the past 4 hours (magenta). Cell leaving the cluster is marked with an asterisk (white). Box plots (a,b) present the median

and 25th and 75th percentiles, and the lower and upper boundaries of whiskers show the 5th and 95th percentiles. Scale bars (f), 50 µm. Data source and statistical details are provided as a source data file.

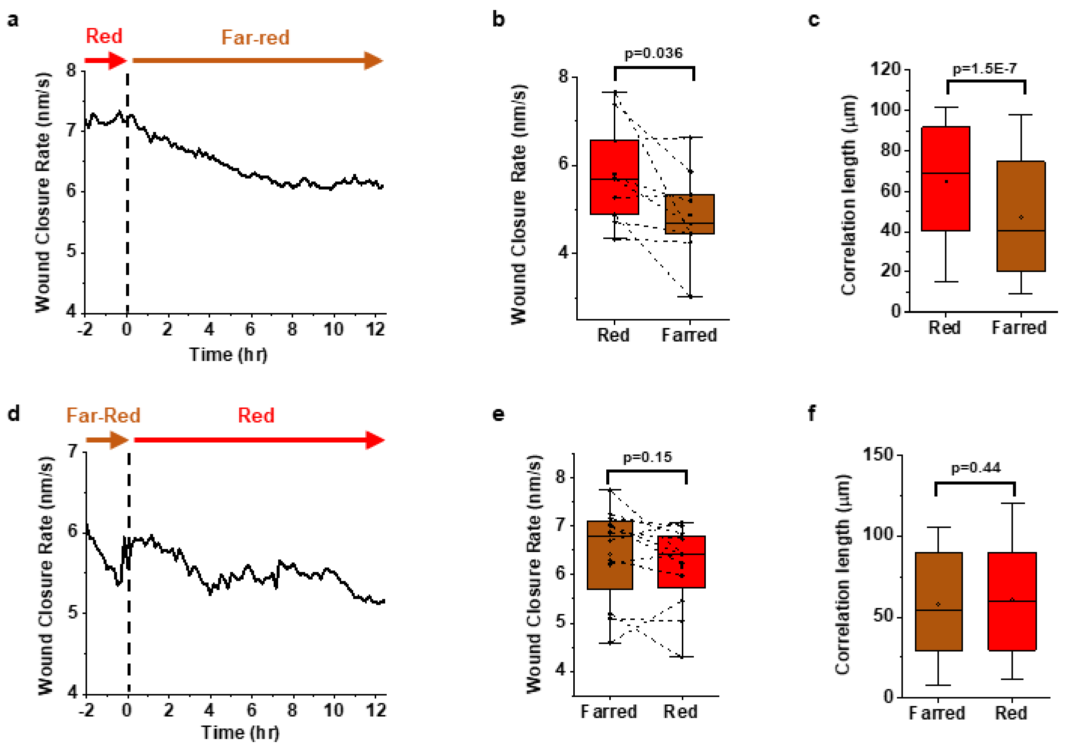

Supplementary Figure 4. Photoregulation of Cph1-based cell-cell adhesions in collective and single-cell migration. (a) Changes in the wound closure rate of Cph1-PM-MDA cells over time on exposure to red light (-6 to 0 hours) and far-red light (0 to 12 hours). Since cell movement during the first 4 hours was unstable, we excluded the data from -6 to -4 hours in the wound closure rate. **(b)** The average wound closure rate of Cph1-PM-MDA cells from -2 to 0 hours under red light and 8 to 10 hours under far-red light (n=8, 2 biological replicates, two-tailed, paired sample t-test). **(c)** Correlation length of Cph1-PM-MDA cells from -6 to 0 hours under red (n=215) light and from 0 to 6 hours under far-red (n=144) light from 2 biological replicates (two-tailed, two-sample Mann-Whitney test). **(d)** Changes in the wound closure rate of Cph1-PM-MDA cells over time when exposed to far-red light (-6 to 0 hours) and red light (0 to 12 hours). **(e)** The average wound closure rate of Cph1-PM-MDA cells from -2 to 0 hours under far-red light and from 8 to 10 hours under red light (n=8, 2 biological replicates, two-tailed, paired sample t-test). **(f)** Correlation length of Cph1-PM-MDA cells from -6 to 0 hours under far-red (n=314) light and from 0 to 6 hours under red (n=140) light from 2 biological replicates (two-tailed, two-sample Mann-Whitney test). Box plots (b,c,e,f) present the median and 25th and 75th percentiles, and the lower and upper boundaries of whiskers show the 5th and 95th percentiles. Data source and statistical details are provided as a source data file.

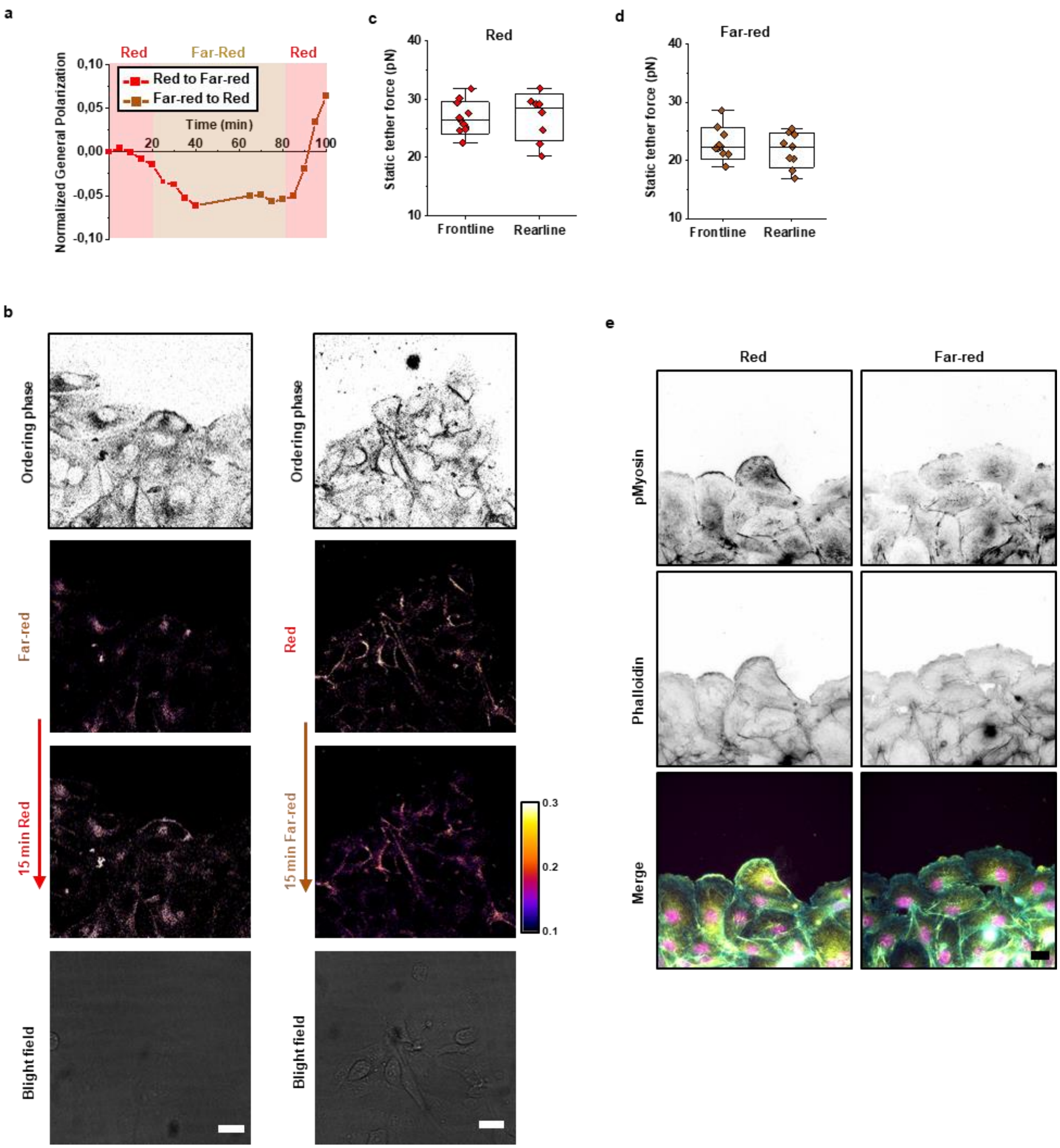

Supplementary Figure 5. Activation of Cph1-PM augments membrane tension and pMyosin expression. (a) The line plot presents the normalized general polarization for the cell-cell interface under both red and far-red light over time. **(b)** Pro12A staining shows polarization changes on switching illumination from far-red to red light and from red to far-red light after wounding. The ordered phase images were used to detect the boundaries of the cells. The bright field images make it difficult to recognize the cell interface. **(c,d)** Static tether forces were measured after exposures to either red or far-red light tension in the frontline and rearline in the wound direction both under red (c) or far-red (d) light (two-tailed, two-sample t-test). **(e)** Fluorescence images of Cph1-PM-MDA cells stained with phalloidin (cyan), anti-pMyosin antibody (yellow), and DAPI (violet). Box plots (c,d) present the median and 25th and 75th percentiles, and the lower and upper boundaries of whiskers show the 5th and 95th percentiles. Data source and statistical details are provided as a source data file.

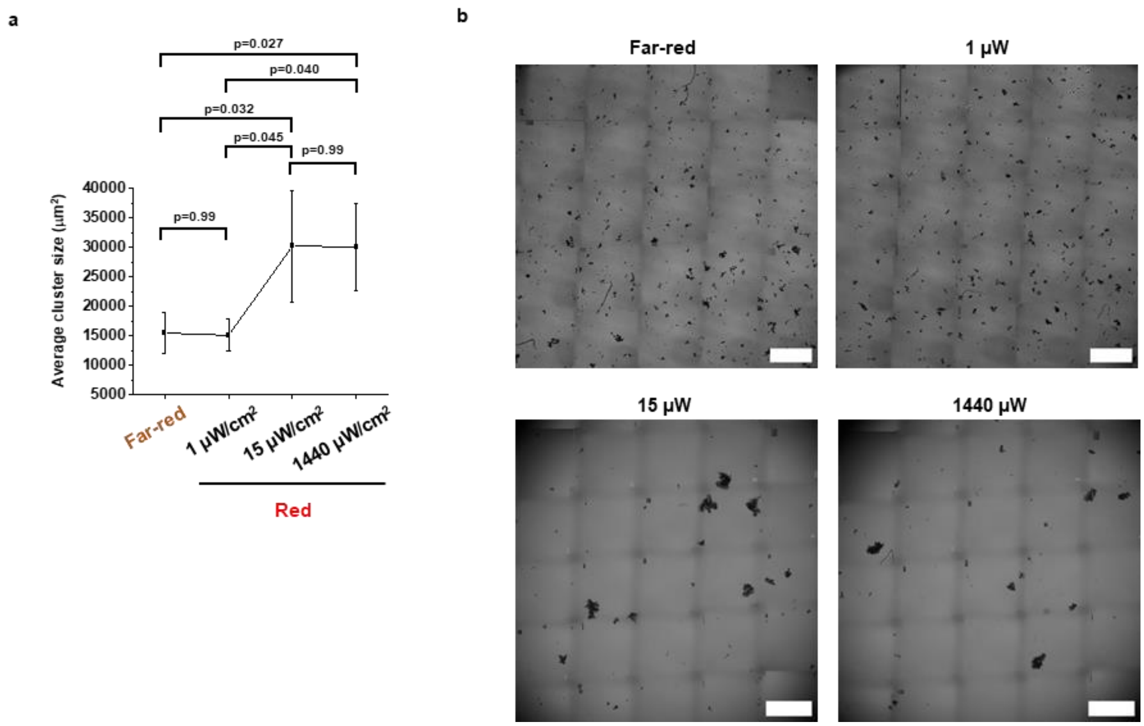

Supplementary Figure 6. Activation of Cph1-PM-based cell-cell adhesions from red light intensity. To determine whether Cph1 expressed in Cph1-PM MDA cells was functional, we used a suspension culture by applying shear stress through a shaker at 60 rpm. This setup allowed for the formation of protein clusters—larger clusters indicate Cph1 protein activation, and smaller clusters suggest Cph1 protein inactivation. **(a)** Average cluster size of Cph1-PM-MDA cells under far-red light (n=4), 1 µW/cm² red light (n=3), 15 µW/cm² red light (n=5), and 1440 µW/cm² red light (n=6) from 2 biological replicates (one-way ANOVA with Tukey's multiple comparisons test). **(b)** Bright field images of Cph1-PM-MDA by varying the light intensity of red light. Scale bars are 1 mm. Line plots (a) present the mean with standard deviation. Data source and statistical details are provided as a source data file.

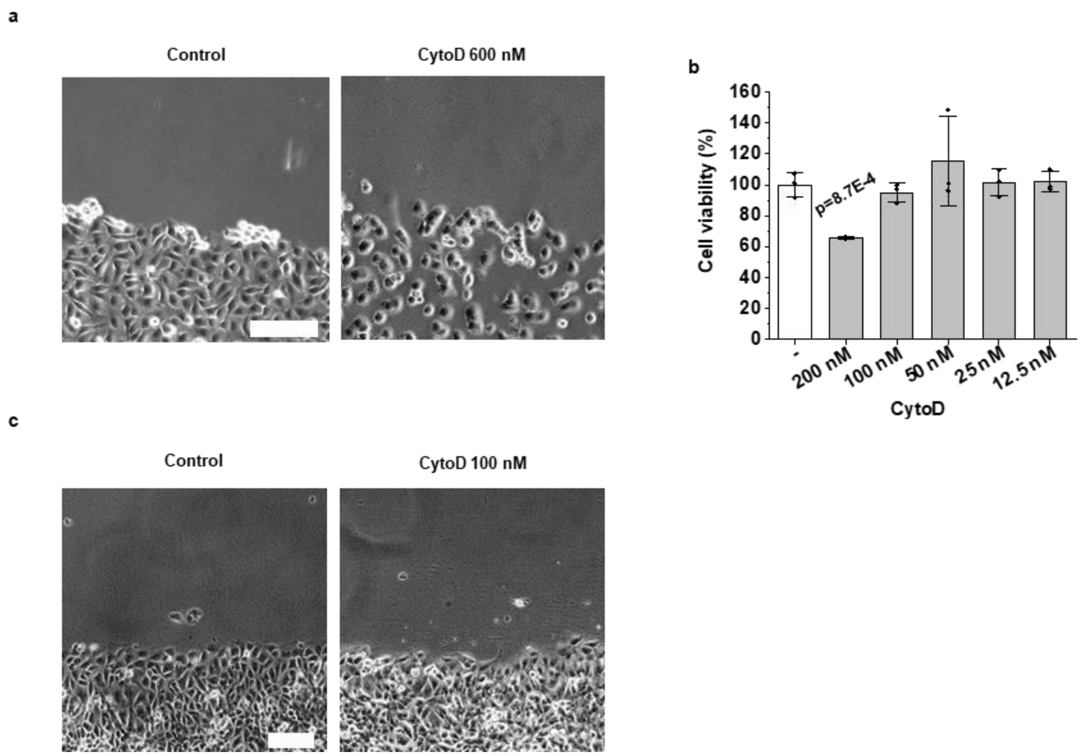

Supplementary Figure 7. Cph1-PM-MDA viability at different CytoD concentrations. (a) Cell toxicity observed with the CytoD inhibitor at a concentration of 600 nM. **(b)** The 3-(4,5-dimethylthiazol-2-yl)−2,5-diphenyltetrazolium bromide (MTT) assay was used to determine a suitable CytoD concentration that was not toxic to the cells (n=3, two-tailed, two-sample t-test). **(c)** Bright field images of cells after addition of 100 µM of CytoD. Bar plot (b) denotes the mean with standard deviation. Scale bars (a,c), 100 µm. Data source and statistical details are provided as a source data file.

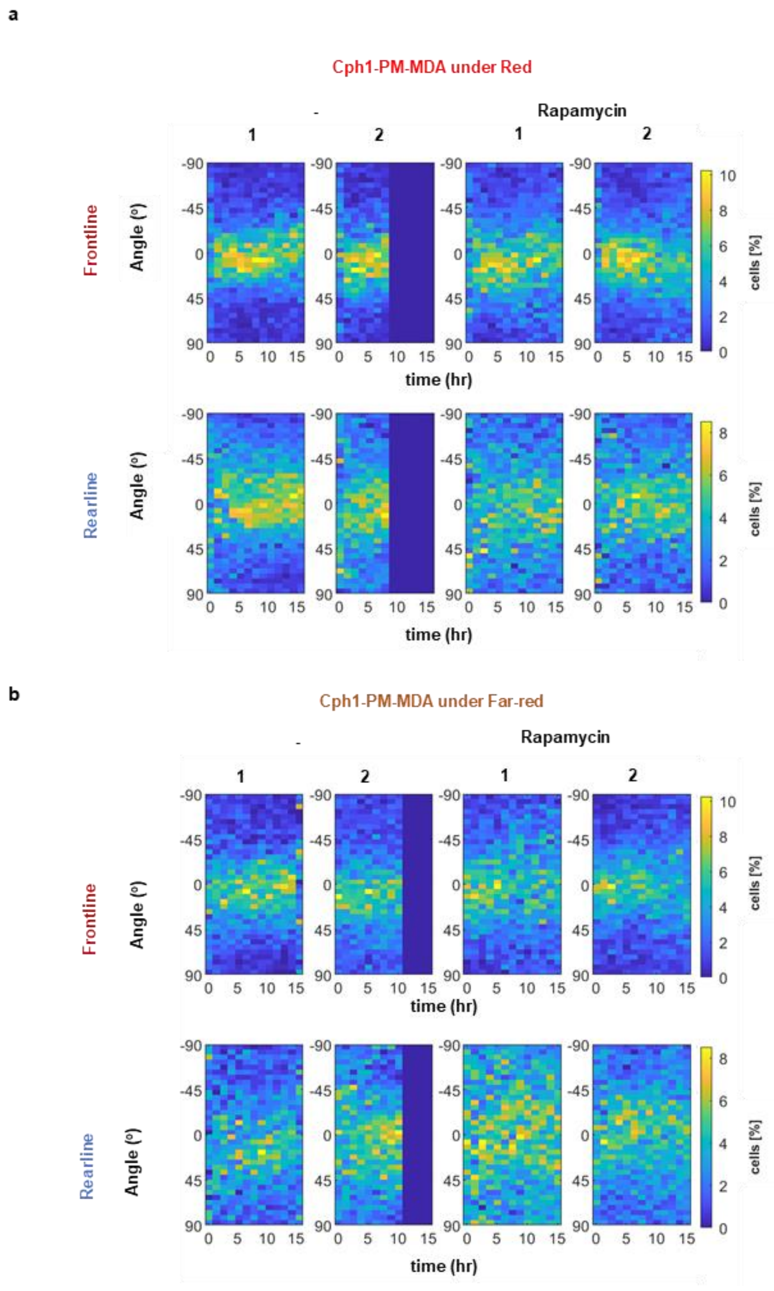

Supplementary Figure 8. Migration angles of Cph1-PM-MDA cells without or with rapamycin. (a, b) Plots showing the migration angle of individual Cph1-PM-MDA cells under red (a) and far-red (b) light. For both conditions, cells in the frontlines (top panel) and rearlines (bottom panel) are shown without treatment (left 2 panels) as well as on addition of rapamycin (right 2 panels). The number indicates the technical replicates. Color code is uniformly scaled for all experiments at the frontline (top panels) and at the rearline (bottom panel), as indicated to the right.

Supplementary Table 1: Sequences and melting temperatures of primers used for gene expression analysis.

Target gene	Sequences	Tm (ºC)	Product size
ZEB1	F: GCACAACCAAGTGCAGAAGA	58.98	141 bp
	R: GCCTGGTTCAGGAGAAGATG	57.96	
FN1	F: GGAGCAAATGGCACCGAGATA	60.48	132 bp
	R: AGCTGCACATGTCTTGGGAA	59.89	
TEAD1	F: GATTCAAACAGGGCAGCCAG	59.47	185 bp
	R: AAAATTCCACCAGGCGAAGC	59.40	
CTGF	F: GGCTTACCGACTGGAAGACA	59.39	104 bp
	R: TCCCACAGGTCTTGGAACAG	59.24	
GAPDH	F: CCTGCACCACCAACTGCTTA	60.54	120 bp
	R: GGCCATCCACAGTCTTCTGAG	60.41	

Supplementary Code 1

```
function [C]=CorLength(u_comp,v_comp) %vector u and v components
clc
[L,W]=size(u_comp{1});
unit=%horizontal length /(W+1);
X=zeros(L,W);
Y=zeros(L,W);
C=[];
E=zeros(L,W);
u_meanSub=zeros(length(u_comp),1);
rho=zeros(L,W);

for i=25:length(u_comp)
 [~,rho]=cart2pol(u_comp{i},v_comp{i});
 for j=1:L
     for k=1:W
         if abs(rho(j,k))< %lower threshold to remove errant vectors
             u_comp{i}(j,k)=NaN;
         end
     end
 end
 u_meanSub(i)=nanmean(nanmean(u_comp{i}));
end
u_mean=nanmean(u_meanSub);

for t=30:length(u_comp)-6
     u_compSub=((u_comp{t-6}+u_comp{t-5}+u_comp{t-4}+u_comp{t-3}+u_comp{t-2}+u_comp{t-
1}+u_comp{t}+u_comp{t+1}+u_comp{t+2}+u_comp{t+3}+u_comp{t+4}+u_comp{t+5}+u_comp{t+6})/13)-
u_mean;
    for i=1:L
        for j=1:W
```

```
            for k=1:L
                for m=1:W

Out=((u_compSub(i,j)).*(u_compSub(k,m)))/sqrt(((u_compSub(i,j))^2).*((u_compSub(k,m))^2));
                    if Out<0.1
                        R=abs(cart2pol(i-k,j-m))*unit;
                        if R<E(i,j)&&E(i,j)~=0
                            E(i,j)=R;
                        elseif E(i,j)==0
                            E(i,j)=R;
                        end
                    end
                end
            end
            if E(i,j)==0
                E(i,j)=NaN;
            end
        end
    end
    C=cat(1,C,E(:));
end
C=rmmissing(C);
end
```

Supplementary Code 2

```
function rho=ColoredVectors(u,v)% input vector components from single frame

clc

[L,W]=size(u);

[~,rho]=cart2pol(u,v);

Rmax=max(max(rho));

f=figure;
set(f,'Color','k')
hold on
for i=1:L
        for j=1:W
            color=(rho(i,j)/Rmax);
            U_sub=zeros(L,W);
            V_sub=zeros(L,W);
            if abs(rho(i,j))<3E-9
                u(i,j)=0;
                v(i,j)=0;
            else
                U_sub(i,j)=u(i,j);
                V_sub(i,j)=v(i,j);
                q=quiver(U_sub,V_sub,'color',[(abs(1-2*(1-color))+(1-2*(1-color)))/2,-abs(2*color-1)+1,(abs(1-
2*color)+(1-2*color))/2],'linewidth',3);
                q.AutoScaleFactor = color;
```

```
            end
        end
end
    axis off
    hold off
    set(q,'Color','k')
end
```

Supplementary Code 3:

```
function vectorPlot(u_filtered,v_filtered)

clc

rhoString=zeros(1,1);
thetaString=zeros(1,1);
thetaComp=zeros(1,1);
for j=1:length(u_filtered)
    [theta,rho]=cart2pol(u_filtered{j},v_filtered{j});
    theta=reshape(theta.',1,[]);
    rho=reshape(rho.',1,[]);
    theta=theta-pi()/2;
    rhoString=[rhoString -rho];
    thetaString=[thetaString theta];
    for i=1:length(theta)
        if theta(i)<0
            theta(i)=theta(i)+2*pi();
        end
        if theta(i)>pi()&&theta(i)<3/2*pi()
            theta(i)=theta(i)-pi()/2;
        elseif theta(i)>3/2*pi()
            theta(i)=theta(i)+pi/2;
        end
        theta(i)=pi()-theta(i);
        thetaComp=[thetaComp theta(i)];
    end
end
thetaString(1)=[];
rhoString(1)=[];
thetaComp(1)=[];
for i=1:length(rhoString)
    if rhoString(i)==0
        rhoString(i)=nan;
    end
end
figure
    h=polarhistogram(thetaComp,12,'FaceColor', % color);
    f=gca;
    f.ThetaLim=[0 180];
    thetaticks([0 45 90 135 180])
    thetaticklabels({'0^o','45^o','90^o','135^o','180^o'})
    ax = ancestor(h, 'polaraxes');
    ax.ThetaZeroLocation = 'top';
```

```
    ax.ThetaDir = 'clockwise';
    f.FontSize=20;
    f.FontWeight='bold';
    rlim([% lower and upper limits]);
    rticks([% ticks])
    rticklabels({% labels})
    ax.RAxis.Label.String='# of vectors';
end
```

Supplementary Code 4:

```
selectImage("Blue"); //Blue channel:445nm (Ordering)
run("32-bit");
selectImage("Green");//Green channel:525nm (Disordering)
run("32-bit");
imageCalculator("Substract create", "Green","Blue");
imageCalculator("Add create", "Blue","Green");
selectImage("Result of Blue");

selectImage("Result of Blue");
run("16-bit");
run("32-bit");
selectImage("Result of Green");
run("16-bit");
run("32-bit");
selectImage("Mask") //Generate "Mask" image by cell masks using Blue channel
run("Divide...", "value=2.5");
imageCalculator("Divide create", "Result of Blue","Result of Green");
selectImage("Result of Result of Blue");
imageCalculator("Multiply create", "Mask","Result of Result of Blue");
selectImage("Result of Mask");
run("Fire");
```
