# Supplementary material for: Intercellular adhesion boots collective cell migration through elevated membrane tension
Source: Nat Commun. 2025 Feb 12;16:1588. doi: 10.1038/s41467-025-56941-4 (PMC11822051; doi:10.1038/s41467-025-56941-4)
Supplement: Supplementary file 2 — Description of Additional Supplementary Information [file 41467_2025_56941_MOESM2_ESM.docx]

**Description of Additional Supplementary Files**

File Name: Supplementary Movie 1

Description: Cellular migration for wound-healing assay of MDA and MCF-7 cells. Frame rate (20 fps, with 20 frames per second). Scale-bar: 100 μm.

File Name: Supplementary Movie 2

Description: Cellular migration for wound-healing assay of Cph1-PM-MDA cells under far-red and red light. Frame rate (20 fps, with 20 frames per second). Scale-bar: 100 μm

File Name: Supplementary Movie 3

Description: Single-cell tracking of individual cell nuclei of Cph1-PM-MDA cells under far-red or red light. Frame rate (20 fps, with 20 frames per second). Scale-bar: 100 μm.

File Name: Supplementary Movie 4

Description: Cellular migration for wound-healing assay of Cph1-PM-MDA cells under red light for 6 hours and then under far-red light for 12 hours. Frame rate (20 fps, with 20 frames per second). Scale-bar: 100 µm.

File Name: Supplementary Movie 5

Description: Cellular migration for wound-healing assay of Cph1-PM-MDA cells under far-red light for 6 hours and then under red light for 12 hours. Frame rate (20 fps, with 20 frames per second). Scale-bar: 100 µm.

File Name: Supplementary Movie 6

Description: Cellular migration for wound-healing assay of Cph1-PM-MDA cells under far-red or red light in the presence of VU. Frame rate (20 fps, with 20 frames per second). Scale-bar: 100 µm

File Name: Supplementary Movie 7

Description: Cellular migration for wound-healing assay of Cph1-PM-MDA cells under far-red or red light in the presence of Oleic acid. Frame rate (20 fps, with 20 frames per second). Scale-bar: 100 µm.
