# Supplementary material for: Intercellular adhesion boots collective cell migration through elevated membrane tension
Source: Nat Commun. 2025 Feb 12;16:1588. doi: 10.1038/s41467-025-56941-4 (PMC11822051; doi:10.1038/s41467-025-56941-4)
Supplement: Supplementary file 10 — Reporting Summary [file 41467_2025_56941_MOESM10_ESM.pdf]

nature portfolio

Corresponding author(s): Seraphine V. Wegner

Last updated by author(s): 2024/01/27

Reporting Summary

Nature Portfolio wishes to improve the reproducibility of the work that we publish. This form provides structure for consistency and transparency in reporting. For further information on Nature Portfolio policies, see our Editorial Policies and the Editorial Policy Checklist.

Statistics

For all statistical analyses, confirm that the following items are present in the figure legend, table legend, main text, or Methods section.

n/a	Confirmed	
☐	☒	The exact sample size (*n*) for each experimental group/condition, given as a discrete number and unit of measurement
☐	☒	A statement on whether measurements were taken from distinct samples or whether the same sample was measured repeatedly
☐	☒	The statistical test(s) used AND whether they are one- or two-sided<br>*Only common tests should be described solely by name; describe more complex techniques in the Methods section.*
☒	☐	A description of all covariates tested
☐	☒	A description of any assumptions or corrections, such as tests of normality and adjustment for multiple comparisons
☐	☒	A full description of the statistical parameters including central tendency (e.g. means) or other basic estimates (e.g. regression coefficient) AND variation (e.g. standard deviation) or associated estimates of uncertainty (e.g. confidence intervals)
☐	☒	For null hypothesis testing, the test statistic (e.g. *F*, *t*, *r*) with confidence intervals, effect sizes, degrees of freedom and *P* value noted<br>*Give P values as exact values whenever suitable.*
☒	☐	For Bayesian analysis, information on the choice of priors and Markov chain Monte Carlo settings
☒	☐	For hierarchical and complex designs, identification of the appropriate level for tests and full reporting of outcomes
☒	☐	Estimates of effect sizes (e.g. Cohen's *d*, Pearson's *r*), indicating how they were calculated

Our web collection on statistics for biologists contains articles on many of the points above.

Software and code

Policy information about availability of computer code

Data collection	Microscopy images were acquired on Leica microscopes with the Leica Application Suite X Ver. 3.7.1.21655.
Data analysis	MATLab R2020a Ver. 9.8.0.1323502 was used to analyze correlation length, colored vector plot. All image analysis was done using ImageJ version 1.53f51 as described in the Methods. All data analysis and statistical analysis was done with OriginPro 2020 version 9.7.0.185. FACS data was visualized with FlowJo Ver. 10.8.1. Ill image analysis was done using ImageJ version 1.53f51 as described in the Methods.

For manuscripts utilizing custom algorithms or software that are central to the research but not yet described in published literature, software must be made available to editors and reviewers. We strongly encourage code deposition in a community repository (e.g. GitHub). See the Nature Portfolio guidelines for submitting code & software for further information.

Data

Policy information about availability of data

All manuscripts must include a data availability statement. This statement should provide the following information, where applicable:

- Accession codes, unique identifiers, or web links for publicly available datasets
- A description of any restrictions on data availability
- For clinical datasets or third party data, please ensure that the statement adheres to our policy

All data in this study are available. Source data are also provided with this study. Primary imaging data of large size will be available for research purposes upon request, provided within 4 weeks and accessible for the next 10 years.

Research involving human participants, their data, or biological material

Policy information about studies with human participants or human data. See also policy information about sex, gender (identity/presentation), and sexual orientation and race, ethnicity and racism.

Reporting on sex and gender	Does not apply
Reporting on race, ethnicity, or other socially relevant groupings	Does not apply
Population characteristics	Does not apply
Recruitment	Does not apply
Ethics oversight	Does not apply

Note that full information on the approval of the study protocol must also be provided in the manuscript.

Field-specific reporting

Please select the one below that is the best fit for your research. If you are not sure, read the appropriate sections before making your selection.

[x] Life sciences [ ] Behavioural & social sciences [ ] Ecological, evolutionary & environmental sciences

For a reference copy of the document with all sections, see nature.com/documents/nr-reporting-summary-flat.pdf

Life sciences study design

All studies must disclose on these points even when the disclosure is negative.

Sample size	The sample sizes are included in the manuscript. The minimum number of samples was chosen such that they reflected statistical significance and reflected the variation between experiments.
Data exclusions	No data was excluded.
Replication	All experiments include multiple technical replicates with at least 2 different biological replicates (experiments on different days). All replicates were successful and the pooled data was included in the analysis.
Randomization	Samples subjected to different treatments were randomly assigned.
Blinding	Does not apply, as the same person has carried out the treatment on the cells and the subsequent analysis.

Reporting for specific materials, systems and methods

We require information from authors about some types of materials, experimental systems and methods used in many studies. Here, indicate whether each material, system or method listed is relevant to your study. If you are not sure if a list item applies to your research, read the appropriate section before selecting a response.

Materials & experimental systems

n/a	Involved in the study	
[ ]	[x]	Antibodies
[ ]	[x]	Eukaryotic cell lines
[x]	[ ]	Palaeontology and archaeology
[x]	[ ]	Animals and other organisms
[x]	[ ]	Clinical data
[x]	[ ]	Dual use research of concern
[x]	[ ]	Plants

Methods

n/a	Involved in the study	
[x]	[ ]	ChIP-seq
[x]	[ ]	Flow cytometry
[x]	[ ]	MRI-based neuroimaging

Antibodies

Antibodies used	All antibodies are commercially available and included in the methods with cat#. Mouse-anti-myc-tag monoclonal antibody (#2276, Cell Signaling), rabbit-anti-YAP antibody (#14074, Cell signaling), rabbit-anti-pERM antibody (#3726, Cell Signaling), mouse- anti-Vinculin monoclonal antibody (13-9777-82, Invitrogen), Alexa 488 anti-rabbit (#4412, Cell Signaling), Alexa 488 anti-mouse (#A11029, Invitrogen), mouse-anti-E-cadherin antibody (#14472, Cell Signaling), 1:1000 rabbit-anti-Vimentin antibody (#5741, Cell Signaling), rabbit-anti-β-actin antibody (#4970, Cell Signaling), HRP-based anti-mouse secondary antibody (1:1000, #7076, Cell signaling), HRP-

	based anti-rabbit secondary antibodies (#7074, Cell Signaling)
Validation	Validated by the manufacturer.<br>Mouse-anti-myc-tag monoclonal antibody (#2276, Cell Signaling) reactive to all the species but sensitive only transfected protein. The antibody can be used all the experiments, including Flow Cytometry, Western blot, and Immunostaining.<br>abbit-anti-YAP antibody (#14074, Cell signaling) reactive to Human, Mouse, Rat, Hamster, and Monkey in Western Blotting, Immunoprecipitation, Immunohistochemistry, Immunofluorescence, Flow Cytometry, Chromatin Immunoprecipitation, and CUT & RUN.<br>rabbit-anti-pERM antibody (#3726, Cell Signaling) reactive to Human, Mouse, Rat, and Monkey for use in Western blot, Immunofluorescence and Immunohistochemical analysis.<br>mouse-anti-Vinculin monoclonal antibody (13-9777-82, Invitrogen) reactive to Human, Mouse and Rat for use in Western blot, Immunohistochemistry, and Immunofluorescence.<br>mouse-anti-E-cadherin (cell signalling # 14472) reactive to Human, Mouse and Rat for use in Western blot, Immunofluorescence and Flow Cytometry.<br>1:1000 rabbit-anti-Vimentin antibody (#5741, Cell Signaling)reactive to Human, Mouse, Rat, Hamster, and Monkey in Western Blotting, Immunohistochemistry, Flow Cytometry, and Immunofluorescence.<br>mouse-anti-β-actin (Abcam, #ab49900) reactive to Mouse, Rat, Hamster, Cow, Dog, Human, African green monkey β-actin for use in Western blot.

Eukaryotic cell lines

Policy information about cell lines and Sex and Gender in Research

Cell line source(s)	MDA-MB-231 and MCF7 were purchased from ATCC. Cph1-PM-MDA was generated and validated in this study
Authentication	MDA-MB-231 (STR profiling) was validated by ATCC.
Mycoplasma contamination	Cells were tested regularly for mycoplasma contamination and results were always negative.
Commonly misidentified lines (See ICLAC register)	Does not apply.

Plants

Seed stocks	*Report on the source of all seed stocks or other plant material used. If applicable, state the seed stock centre and catalogue number. If plant specimens were collected from the field, describe the collection location, date and sampling procedures.*
Novel plant genotypes	*Describe the methods by which all novel plant genotypes were produced. This includes those generated by transgenic approaches, gene editing, chemical/radiation-based mutagenesis and hybridization. For transgenic lines, describe the transformation method, the number of independent lines analyzed and the generation upon which experiments were performed. For gene-edited lines, describe the editor used, the endogenous sequence targeted for editing, the targeting guide RNA sequence (if applicable) and how the editor was applied.*
Authentication	*Describe any authentication procedures for each seed stock used or novel genotype generated. Describe any experiments used to assess the effect of a mutation and, where applicable, how potential secondary effects (e.g. second site T-DNA insertions, mosiacism, off-target gene editing) were examined.*
